# Duchenne Muscular Dystrophy With Low Acidic α-Glucosidase Activity: Two Case Reports and Literature Review

**DOI:** 10.3389/fped.2022.855510

**Published:** 2022-06-01

**Authors:** Xiufang He, Xuandi Li, Yuese Lin, Hongjun Ba, Huimin Peng, Lili Zhang, Ling Zhu, Youzhen Qin, Shujuan Li

**Affiliations:** ^1^Department of Pediatric Cardiology, Heart Center, The First Affiliated Hospital, Sun Yat-sen University, Guangzhou, China; ^2^Key Laboratory on Assisted Circulation, Ministry of Health, Guangzhou, China

**Keywords:** acid α-glucosidase, pseudodeficiency alleles, Duchenne muscular dystrophy, creatine kinase, child

## Abstract

**Background:**

Pompe disease is usually considered in children with elevated creatine kinase (CK) levels and decreased acidic α-glucosidase (GAA) enzyme activity. However, there are exceptions, such as GAA pseudo deficiency alleles, which result in lower GAA enzyme activity but do not cause Pompe disease. Here, we report two cases presenting with high CK levels and low GAA activity who were ultimately diagnosed with Duchenne muscular dystrophy (DMD).

**Case Presentation:**

Case 1 patient was a 2-month-old boy who presented with an extremely high serum CK level (5,480∼11,880 U/L) and low GAA activity (2.72 nmol/1 h/mg). The whole-exome sequencing did not find the pathogenic GAA gene mutation, however, there was a DMD gene hemizygous variation (c. 7657C > T, p. Arg2553Ter) inherited from his mother, which was verified by the first-generation sequencing. Further genetic analysis of GAA identified two homozygous pseudo deficiency alleles (c.1726G > A, p. Gly576Ser and c.2065G > A, p. Glu689Lys), which were believed to induce the patient’s low GAA activity. Therefore, the boy was diagnosed with DMD, although he had extremely low GAA activity. Case 2 patient was also a 2-month-old boy presenting with a significant increase in CK level (12,408∼24,828 U/L). His blood GAA activity (colorimetric method) was 9.02 nmol/1 h/mg. Similarly, his whole-exome sequencing did not find the pathogenic mutation of the GAA gene, but a DMD gene hemizygous variation (c.5571del, p. Lys1857AsnfsTer8), hence he was diagnosed with DMD as well. Regarding GAA activity, the case 2 patient was not as low as the case 1 patient, mainly because his two GAA pseudo deficiency alleles were heterozygous.

**Conclusion:**

Pompe disease is usually screened in infants with high CK levels. We should be aware that pseudo deficiency alleles can cause low GAA activities but not Pompe disease. Genetic tests would be helpful to distinguish cases with GAA pseudo deficiency alleles from patients with some muscular disorder diseases such as DMD.

## Introduction

Neuromuscular diseases such as Duchenne muscular dystrophy (DMD) and some glycogen storage diseases like Pompe disease are considered in children having high blood creatine kinase (CK) levels.

The DMD is an X-linked recessive genetic disease caused by a mutation in the gene for the protein dystrophin. Dystrophin is important to maintain the muscle fiber’s cell membrane. Lack of dystrophin causes progressive muscle weakness. CK levels in the bloodstream are usually extremely high. Genetic testing (DNA testing) and a muscle biopsy test confirm the diagnosis in most cases (1).

Pompe disease, also called Glycogen storage disease type II, is an autosomal recessive metabolic disorder caused by an accumulation of glycogen in the lysosome due to deficiency of the lysosomal acid alpha-glucosidase (GAA) enzyme, which is caused by a mutation in the GAA gene. The build-up of glycogen causes progressive muscle weakness, particularly in the skeletal muscles and the heart. Typical findings are those of an enlarged heart in the early-onset form and gradually progressive muscle weakness in the late-onset form. Serum CK levels are high. Diagnosis is made by estimating the GAA activity and genetic testing (2). However, due to the pseudo deficiency alleles, the GAA activity levels might be false positive (3).

The prevalence of Pompe disease is about 2 in 100,000. On the other hand, the prevalence of DMD in males is about 10 in 100,000 while in females is less than 0.1 per 100,000. Regarding the clinical features, these two diseases are overlapped. Thus, clinicians need to be careful in differential diagnosis, especially when facing infants with high CK levels. The major treatment for Pompe disease is enzyme replacement therapy, while it needs multidisciplinary management in DMD without a favorable cure (2, 4). Since the treatment and prognosis of the two diseases are different, we highlight the accurate diagnosis and differential diagnosis, and every case report for reference.

In this article, we report two infants with high CK levels and low GAA activity who were initially presumed to have Pompe disease. However, their genetic testing identified DMD gene mutation and GAA pseudo deficiency alleles, so they were diagnosed with DMD eventually. We present these two cases to emphasize that when facing a patient with increased CK and decreased GAA activity, clinicians should be aware of the GAA pseudo deficiency alleles and consider the diagnosis cautiously.

## Case Presentation

### Case 1

**FIGURE 1:**
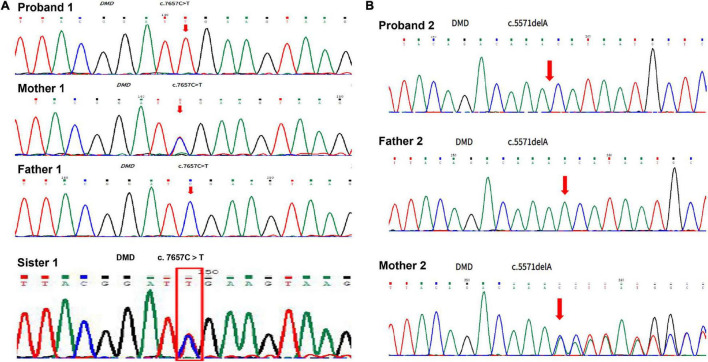
Duchenne muscular dystrophy DMD gene sequencing for the patients and their lineal relatives. **(A)** Result of DMD gene sequencing in case 1. **(B)** Result of DMD gene sequencing in case 2.

A 2-month-old baby boy, who was born at 38 weeks of gestational age and weighed 3,800 g, had a blood test because of jaundice and accidentally found a significantly increased CK level. He had no family history of muscular dystrophy. He got no respiratory distress, muscle weakness, feeding difficulties, failure to thrive, or other positive findings. The muscle tone detection revealed fine. The Motor Assessment of the Developing infant (AIMS) showed his motor development as normal, with a score of 11 as a 2-month-old baby. His blood tests revealed extremely high CK levels of 5,480∼11,880 U/L with lesser elevations of alanine transaminase (ALT 197 U/L), aspartate transaminase (AST 455 U/L), and lactic dehydrogenase (LDH 788 U/L). Due to the high CK level, GAA activity was tested and found an extremely low level of 2.72 nmol/1 h/mg (colorimetric method, reference value of >14 nmol/1 h/mg). Urine organic acids, dry filter paper blood spot amino acid, and acylcarnitine analysis had no positive findings. This boy was supposed to have Pompe disease according to the above findings. However, the boy did not have either sucking or swallowing difficulties, poor muscle tone, or swelling tongue as those of early-onset form. About 1 week later, whole-exome sequencing was carried out and the result showed that he did not have pathogenic mutations of the GAA gene but a DMD gene hemizygous variation (c. 7657C > T, p. Arg2553Ter) was identified. First-generation sequencing verified his hemizygous variation was inherited from his mother who had a heterozygous variation (Figure 1A and Supplementary Data Sheet). Concurrently, we found that he had two GAA homozygous pseudo deficiency alleles (c.1726G > A, p. Gly576Ser and c.2065G > A, and p. Glu689Lys). These findings supported the diagnosis of DMD and explained why his GAA activity was low. Since the patient has DMD, we checked the CK levels for his family and found that his mother (37 years old) and his sister (8 years old) had asymptomatic elevated CK, which were 437 and 1,069 U/L, respectively. Additionally, the GAA activity testing was performed for the family as well, and the results were shown in Table 1. The parents and sister both had decreased GAA activity, and their GAA genetic testing showed pseudo deficiency alleles as well.

**TABLE 1:** Comparison of acidic α-glucosidase (GAA) activity and GAA variants between probands and their family.

	GAA activity (colorimetric method) nmol/1 h/mg	GAA variants	Homozygote or heterozygote
Proband 1	2.72	c.1726G > A	Homozygote
		c.2065G > A	Homozygote
Mother 1	11.54	c.1726G > A	Heterozygote
		c.2065G > A	Heterozygote
Father 1	8.90	c.1726G > A	Heterozygote
		c.2065G > A	Heterozygote
Sister 1	5.51	c.1726G > A	Homozygote
		c.2065G > A	Homozygote
Proband 2	9.02	c.1726G > A	Heterozygote
		c.2065G > A	Heterozygote
Mother 2	10.46	c.1726G > A	Heterozygote
		c.2065G > A	Heterozygote
Father 2	21.22	c.1726G > A	Wild type
		c.2065G > A	Wild type

### Case 2

A boy, born at 39 weeks of gestational age and weighed 3,200 g, was hospitalized because of neonatal pneumonia and found significantly increased CK, ranging from 3,400 to 3,995 U/L. At 2 months old, he was admitted to our hospital to re-check his CK level. He had no family history of muscular dystrophy and no positive physical findings. The muscle tone and motor development were normal for his age, with a score of 11 in the AIMS motor assessment. Blood tests showed CK of 12,408∼24,828 U/L, ALT of 168 U/L, AST of 253 U/L, and LDH of 973 U/L. A low blood GAA activity was found at 9.02 nmol/1 h/mg (reference value of >14 nmol/1 h/mg). Urine organic acids, dry filter paper blood spot amino acid, and acylcarnitine analysis showed negative results. Based on these findings, the patient was supposed to have Pompe disease. However, his GAA activity was not extremely low, so we performed the whole-exome sequencing 1 week later, which found that he had a DMD gene hemizygous mutation (NM_004006.2: c.5571del, p. Lys1857AsnfsTer8) and two GAA heterozygous pseudo deficiency alleles (c.1726G > A, p. Gly576Ser and c.2065G > A, and p. Glu689Lys), but had no GAA pathogenic mutations. First-generation sequencing verified his DMD gene hemizygous variation which was inherited from his mother who had a heterozygous variation (Figure 1B and Supplementary Data Sheet). The two GAA heterozygous pseudo deficiency alleles explained why his GAA activity was low. Finally, he was diagnosed with DMD. We checked the CK levels and GAA activities of his family and found that his mother (27 years old) had a high CK level of 221U/L and low GAA activity of 10.46 nmol/1 h/mg (Table 1).

### Perception of the Families

At the time when the babies were found CK elevated, they did not present any clinical symptoms. Later, the families were informed that the babies were diagnosed with Pompe disease. Pompe disease could be classified as early-onset and late-onset based on when the clinical features occurred. The families were worried about the onset time, especially the infantile type, which would damage the myocardium, and lead to heart failure or respiratory failure rapidly before the baby turned 1 year old. Weeks later, the gene test confirmed the diagnosis as DMD rather than Pompe disease, so the families were anxious accordingly about the delayed motor ability, incapacity on taking care of themselves, and related psychological problems. Because DMD primarily affected skeletal muscles, it ultimately led to systemic muscle weakness.

In terms of the risk of offspring, Pompe disease was an autosomal recessive disorder, while DMD was X-link recessive disorder. The two diseases were inherited in different ways, resulting in different possibilities of developing the disease in offspring of different genders. Thanks to the correct diagnosis, the families would accept the right guidance in the prenatal examination during the birth of the future offspring.

In terms of treatment management, both diseases affected multi-system that required multidisciplinary integrated treatment. On the one hand, Pompe disease was treatable with enzyme replacement therapy, but it was too expensive for them to afford. On the other hand, there was no effective treatment for DMD at present. The multidisciplinary comprehensive treatment could only try to slow down the progression of the disease, prolong the survival time, and improve their life quality. A correct diagnosis could save them from wasting valuable time and money on enzyme replacement therapy.

Finally, the families fully understood the transition from the initial diagnosis of Pompe disease to the definite diagnosis of DMD. They acknowledged that Pompe disease and DMD were easily confused to some extent because of their overlapping clinical manifestations. With elevated CK levels and low GAA enzyme activity, Pompe disease always came as the first suspect diagnosis. Fortunately, DMD was confirmed by genetic testing. The families were now convinced and accepted the final diagnosis of DMD and have been referred to a neuromuscular specialist for regular follow-up.

## Discussion

The newborn screening for Pompe disease has been carried out in many countries to screen for sometimes-fatal early-onset form and start enzyme replacement therapy earlier. For infants with high CK levels, Pompe disease would be considered, and GAA activity is usually checked. The presence of pseudo deficiency alleles, frequent in Asian populations, might induce false-positive results. Patients with a GAA pseudo deficiency allele show greatly reduced enzyme activity, yet they remain clinically healthy because the enzyme activity is not low enough to display symptoms of Pompe disease. In our report, we presented two cases with high CK levels and low GAA activities, they were confirmed as GAA pseudo deficiency alleles and, finally, were diagnosed as DMD, which had a different clinical spectrum.

Pseudodeficiency alleles can cause a significant reduction in GAA activity, but no symptoms of the disease (5). Pseudodeficiency alleles are known as c.1726G > A (p. Gly576Ser) and c.2065G > A (p. Glu689Lys), which are found more frequently in Asian populations. Newborns with low GAA activity present with 71.8% (102/142) c.1726G > A (p. Gly576Ser) pseudo deficiency alleles and 72.5% (103/142) c.2065G > A (p. Glu689Lys) pseudo deficiency alleles (6). It is reported that substitution c.2065G > A (p. Glu689Lys) reduces GAA activity by 50% at most, while substitution c.1726G > A (p. Gly576Ser) reduces the activity to such extent that it falls into the patient range (7–9). In our two cases, their family members who had low GAA levels all had pseudo deficiency alleles but did not show any symptoms of Pompe disease. Additionally, homozygous pseudo deficiency alleles cause lower GAA activity than heterozygous pseudo deficiency alleles. The frequency of pseudo deficiency alleles in the Japanese population is estimated to be 3.9% for homozygous alleles, whose GAA activity is 1.4–10.1 pmol/punch/h (fluorimetry) and 30.5% for heterozygous alleles, whose GAA activity is about 6.8–58 pmol/punch/h (fluorimetry) (10). Both of our cases had pseudo deficiency alleles, c.1726G > A (p. Gly576Ser) and c.2065G > A (p. Glu689Lys), the one whose GAA activity was 2.72 nmol/1 h/mg had homozygous pseudo deficiency alleles, and the other whose GAA activity was 9.02 nmol/1 h/mg had heterozygous pseudo deficiency alleles. Either a healthy population or patients with Pompe disease could have GAA pseudo deficiency alleles. The frequency of GAA pseudo deficiency alleles in a healthy population and patients with Pompe disease are similar, but homozygous pseudo deficiency alleles occur more frequently in patients with Pompe disease than in a healthy population (10, 11). In the Guangzhou population, the frequencies of pseudo deficiency variants c.1726G > A and c.2065G > A homozygotes are 26.3% (15/57) and 35.1% (20/57) in patients with Pompe disease, which were significantly higher than those [1.7% (40/2 395) and 3.9% (94/2 395)] in healthy children (χ^2^ = 151.2, 121.9; both at *P* < 0.01) (12).

Patients with elevated CK levels also have a high frequency of the GAA pseudo deficiency alleles, in which the muscular disorders should be differentiated from Pompe disease. Lee et al. found that 16 of 90 (17.8%) patients with unspecified myopathy had reduced GAA activity, among which 14 (15.6%) patients had GAA pseudo deficiency alleles and the remaining two patients had late-onset Pompe disease (13). Oitani et al. reported a case that a 3-year-old boy with incidentally detected elevated CK level was initially suspected of Pompe disease because of his low GAA enzyme activity and was ultimately diagnosed with Becker muscular dystrophy (BMD) (14). The patient had no family history of muscular dystrophy or CK elevation. His motor and mental milestones were not delayed. His GAA activity in dried blood spots was 2.50 pmol/punch/h (normal: 21.38 pmol/punch/h) and in lymphocytes was 2.35 nmol/mg protein/h (normal: 30.7 ± 10.3 nmol/mg protein/h). Genetic analysis of GAA detected heterozygosity for a nonsense mutation, c.118C > T (p. Arg40*), and pseudo deficiency alleles, c.1726G > A (p. Gly576Ser) and c.2065G > A (p. Glu689Lys). Skeletal muscle biopsy showed no deposits of glycogen but a significant reduction of dystrophin protein, 11.8% of the normal mean. Direct sequencing of dystrophin revealed hemizygosity for a nonsense mutation, [NM_004006.2: c.72G > A (p. Trp24*)]. Based on these findings, he was diagnosed with BMD eventually. Similarly, our cases were initially suspected of Pompe disease but diagnosed with DMD eventually. This is the first report of DMD with low GAA activity. Overall, due to the pseudo deficiency alleles, reduced GAA activity should be interpreted carefully, especially in patients with elevated CK levels, they might have a different diagnosis.

Muscular dystrophies are a genetically and clinically heterogeneous group of rare neuromuscular diseases that cause the progressive breakdown of skeletal muscles over time. Muscular dystrophies are caused by mutations in genes, usually those involved in making muscle proteins. Of those, DMD accounts for the most common and severe cases, and BMD is relatively milder. According to prior studies, the phenotype of DMD gene mutations that our two patients have is DMD pathogenic variants. DMD gene mutation of the patient 1 was a nonsense mutation (NM_004006.2: c. 7657C > T, p. Arg2553Ter), known as a loss-of-function mutation, which was reported as disease-causing (15–18). DMD gene mutation of patient 2 was a frameshift mutation (NM_004006.2: c.5571del, p. Lys1857AsnfsTer8), also known as a loss-of-function mutation, which was reported as a disease-causing as well (19). Although our two patients were diagnosed with DMD, they had no symptoms of DMD yet. It might largely be because they were only 2 months old that their motor development delays were not obvious enough, which was worth following upon.

The diagnosis of DMD is confirmed by genetic testing (20). Gene diagnosis has become an important role in modern medicine. Thanks to genetic testing, more and more rare diseases can be diagnosed earlier and more accurately. Fortunately, our two cases were accurately diagnosed through genetic testing and had been referred to the neuromuscular specialist timely.

In conclusion, pseudo deficiency alleles can cause a remarkable reduction of GAA activity as in Pompe disease. When a patient has elevated CK and low GAA activity, the diagnosis should be carefully considered, especially in Asian populations, genetic testing is recommended.

## Data Availability Statement

The datasets for this article are not publicly available due to concerns regarding participant/patient anonymity. Requests to access the datasets should be directed to the corresponding authors.

## Ethics Statement

Written informed consent was obtained from the parents for publication of this case report.

## Author Contributions

XH, YQ, and SL conceptualized and designed the study. XH and XL majored in collecting data and drafting the manuscript. HB, HP, and LZha helped to analyze cases and follow up with patients. XH, SL, XL, YL, and LZhu were responsible for the treatment of the patients. All authors read and approved the final manuscript.

## Conflict of Interest

The authors declare that the research was conducted in the absence of any commercial or financial relationships that could be construed as a potential conflict of interest.

## Publisher’s Note

All claims expressed in this article are solely those of the authors and do not necessarily represent those of their affiliated organizations, or those of the publisher, the editors and the reviewers. Any product that may be evaluated in this article, or claim that may be made by its manufacturer, is not guaranteed or endorsed by the publisher.
